# Evaluation safety and efficacy of MRgFUS for palliative treatment of bone metastasis

**DOI:** 10.1186/2050-5736-2-S1-A4

**Published:** 2014-12-10

**Authors:** Vladimir Turkevich, Sergey Kanaev

**Affiliations:** 1 Department radiation oncology and radiology, Petrov Research Institute of Oncology, St. Petersburg, Russian Federation

## Background

The ExAblate system is a non-invasive thermal ablation device that has been used for the ablation of tissue. This system combines a focused ultrasound surgery delivery system and a conventional diagnostic 1.5 T MRI scanner (MRgFUS/MR guided focused ultrasound surgery). Starting from 2009 up to 2012 our Institute participated in the clinical randomized multi-site study to evaluate safety and efficacy of MRgFUS for palliative treatment of Bone Metastasis. This study was approved by FDA at 18 October 2012.

## Material & Methods

In our study were recruited 37 patients with painful bone metastases. 27 patients had a real treatment with the ExAblate® system (*InSightec*, Haifa, Israel) at Petrov Research Institute of Oncology, St. Petersburg, Russia. And 10 patients had a placebo treatment. Immediately after procedure patients were examined for any adverse events and after a brief recovery discharged. Patients were followed up on 1 and 3 days, 1 and 2 weeks, 1, 2 and 3 months post treatment. During each visit, treatment safety was evaluated by recording and assessment of device or procedure related adverse events. Effectiveness of palliation was evaluated using the standard pain scale (0-no pain/10-worst pain imaginable) and by monitoring changes in the intake of pain-relieving medications. A reduction of 2 points or more on pain scale was considered a significant response to treatment. 5 patients were male and 32 female. Mean age was 58 years old (19-79). The primary cancers were: 29 breast, 2 rectum, 1 stomach, 1 bronchus, 1 bladder, 3 other. Targeted lesions were 6 osteolytic 31 mixed. 23 were pelvis metastases, 4 were located in the femoral bone, 2 were located in the upper extremity bones and 8 were located in the ribs.

## Results

No significant device or procedure related adverse events were recorded. 2 patients died during the follow-up period due to disease progression, thus 3 months follow-up data includes only results of 35 patients. All 27 patients from real treatment group were reported significant improvement in pain with no change in their medication intake. Mean worst pain score at baseline, 1 day, 3 days, 1 week, 2 weeks, 1, 2 and 3 months post-treatment was 6.9, 6.1, 5.1, 3.5, 2.6, 1.8, 1.2 and 0.9.

## Conclusions

Results of the study that was performed, palliation effects are significant in terms of mean improvement, the number of treated patients who reported symptomatic improvement and in their potential durability. Based on the above ExAblate treatment has a potential to be treatment of choice for radiation failure patients.

